# CRISPR**–**Cas9-mediated genome editing and guide RNA design

**DOI:** 10.1007/s00335-015-9565-z

**Published:** 2015-05-20

**Authors:** Michael V. Wiles, Wenning Qin, Albert W. Cheng, Haoyi Wang

**Affiliations:** The Jackson Laboratory, 600 Main Street, Bar Harbor, ME 04609-1500 USA; State Key Laboratory of Reproductive Biology, Institute of Zoology, Chinese Academy of Sciences, Beijing, People’s Republic of China

## Abstract

CRISPR and CRISPR-associated (Cas) proteins, which in nature comprise the RNA-based adaptive immune system in bacteria and archaea, have emerged as particularly powerful genome editing tools owing to their unrivaled ease of use and ability to modify genomes across mammalian model systems. As such, the CRISPR**–**Cas9 system holds promise as a “system of choice” for functional mammalian genetic studies across biological disciplines. Here we briefly review this fast moving field, introduce the CRISPR**–**Cas9 system and its application to genome editing, with a focus on the basic considerations in designing the targeting guide RNA sequence.

## Introduction

Site-directed DNA endonucleases are powerful tools for genome editing. When introduced into cells, these proteins can bind to a target DNA sequence in the genome and create a DNA double-strand break (DSB), the repair of which leads to varied DNA sequence modifications. The initial efforts on developing these tools were focused on engineering homing endonucleases (Silva et al. 2011) and zinc finger nucleases (ZFN) (Urnov et al. 2005, 2010), and later Transcription Activator-Like Effector Nucleases (TALEN) (Boch et al. 2009; Moscou and Bogdanove 2009; Bogdanove and Voytas 2011). Homing endonucleases use one single domain to perform both DNA recognition and cleavage functions, and as such, are challenging to engineer. For both the ZFN and TALEN systems, the DNA binding domains (DBD) are modular and can be engineered to recognize and bind specific DNA sequences, allowing an attached nuclease domain to generate DSBs at the target site. However, for each genomic target, a unique pair of ZFN or TALEN needs to be designed and generated, which is cumbersome and time-consuming. In 2012, a novel system, Clustered Regularly Interspaced Short Palindromic Repeats (CRISPR) and the CRISPR-associated proteins (Cas), emerged from the acquired immune system of bacteria and archaea (Jinek et al. 2012). CRISPR**–**Cas9 rapidly became the method of choice for genome editing having many advantages over the earlier approaches (Doudna and Charpentier 2014; Hsu et al. 2014). Here we briefly review this fast moving field, introduce the CRISPR**–**Cas9 system and discuss its application to genome editing, with a focus on the basic considerations in designing the targeting guide RNA sequence.

## CRISPR**–**Cas9-mediated genome editing

The CRISPR**–**Cas system was first described in the genome of *Escherichia coli* as a cluster of short palindromic repeats separated by peculiar short spacer sequences (Ishino et al. 1987). Subsequently, it was shown that CRISPR loci are present in the genomes of more than 40 % of bacteria and 90 % of archaea (Horvath and Barrangou 2010) and their function is to serve as an adaptive immune defense mechanism, protecting against phage infection by recognizing and cleaving pathogen DNA (Horvath and Barrangou 2010; Fineran and Charpentier 2012). By 2012, the basic mechanism of CRISPR**–**Cas9 derived from *Streptococcus pyogenes* was elucidated (Deltcheva et al. 2011; Jinek et al. 2012). CRISPR**–**Cas9 is an RNA-guided DNA endonuclease system in which Cas9 endonuclease forms a complex with two naturally occurring RNA species, CRISPR RNA (crRNA) and trans activating CRISPR RNA (tracrRNA). This complex targets specific DNA sequences complementary to the 20 nt (nucleotide) sequence residing at the 5′ end of the crRNA (Jinek et al. 2012). Conveniently, crRNA and tracrRNA can be linked by an arbitrary stem loop sequence to generate a synthetic single-guide RNA (sgRNA). Although naturally evolving as a system in bacteria, upon appropriate codon optimization of the Cas9 coding sequence, CRISPR–Cas9 is highly active in mammalian cells (Cho et al. 2013; Cong et al. 2013; Jinek et al. 2013; Mali et al. 2013b).

**Fig. 1:**
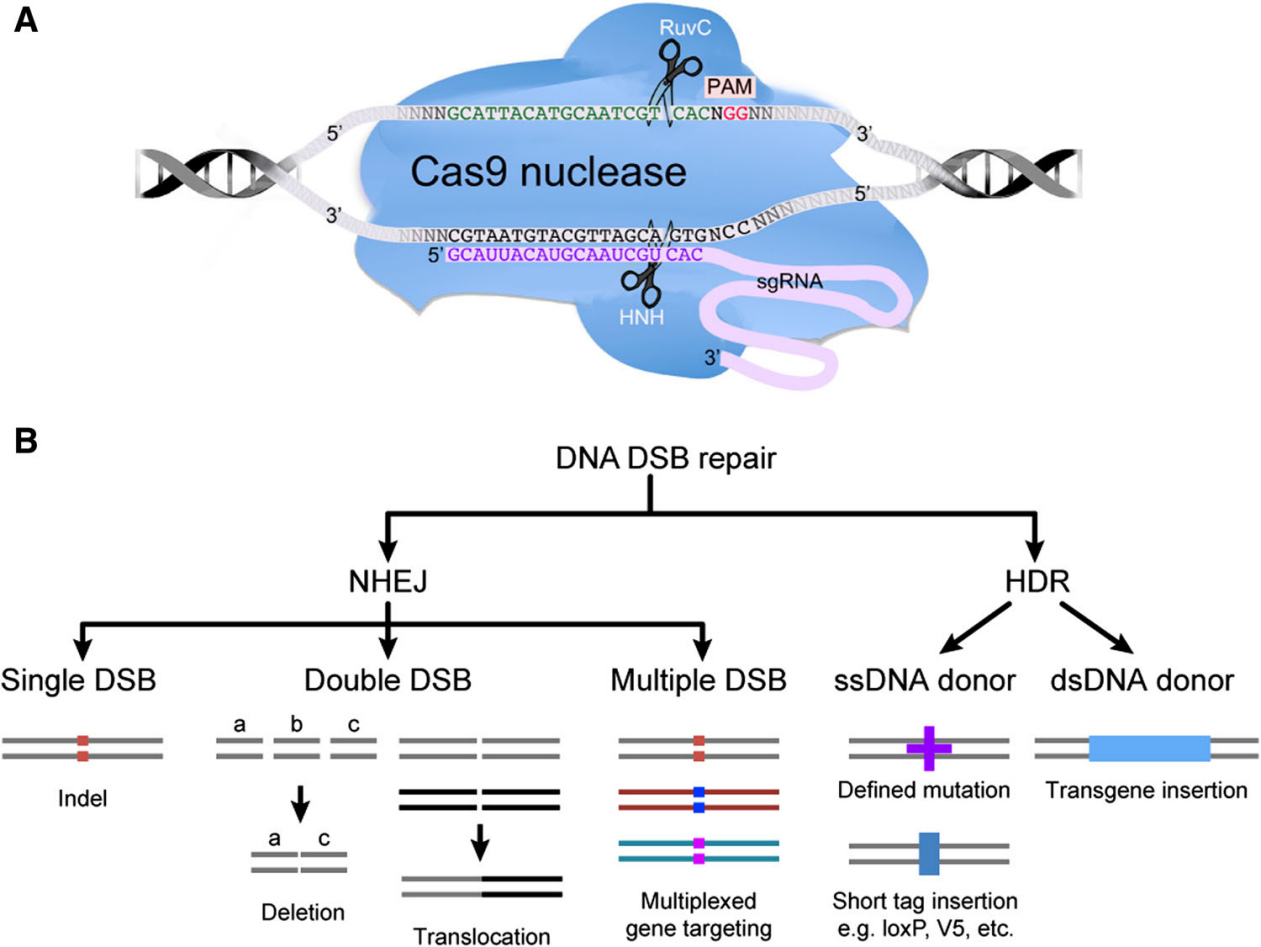
CRISPR–Cas9-mediated genome editing. **a** The structure of Cas9–sgRNA complex binding to target DNA. Cas9 binds to specific DNA sequences via the base-pairing of the guide sequence on sgRNA (*pink*) with the DNA target (*gray*). Protospacer adjacent motif (PAM) is downstream of the target sequence. **b** The CRISPR–Cas9-mediated double-stranded DNA breaks are repaired by endogenous DNA repair machinery: non-homologous end joining (NHEJ) or homology-directed repair (HDR). Various genetic modifications can be generated through these two pathways

In practice, by simply designing the 5′ 20 nt sequence on the sgRNA to be complementary to the genomic target sequence, the Cas9 nuclease-sgRNA complex can be directed to specific genomic locus generating DNA DSBs. The target defining region of the sgRNA is about 20 nt long, with variations from 17 to 30 nt having been successfully used (Ran et al. 2013; Fu et al. 2014). The other key element in determining target sequence specificity is the Protospacer Adjacent Motif (PAM) that is adjacent to the target site at the genome locus, but is *not a part of* the guide RNA sequence (see Fig. 1). For Cas9 nuclease from *S.pyogenes*, the PAM sequence is NGG, while CRISPR–Cas9 systems from other species use different PAM sequences (Cong et al. 2013; Esvelt et al. 2013; Hou et al. 2013). In bacteria, the PAM is thought to effectively distinguish self, with the PAM not being present in the genomic CRISPR loci, from the invading phage, whose genome carries the PAM sequence adjacent to the target sequence (Marraffini and Sontheimer 2010).

CRISPR–Cas9-mediated DNA DSBs are repaired through either the Non-Homologous End Joining (NHEJ) repair process, or the homology-directed repair (HDR) pathway. NHEJ repair often leads to small insertions or deletions (indels) at the targeted site, while HDR pathway leads to perfect repair or precise genetic modification (see Fig. 1) (Doudna and Charpentier 2014; Hsu et al. 2014). Through these two DNA repair pathways, various genetic modifications can be achieved (Fig. 1). The NHEJ-mediated DNA repair pathway can be exploited to generate null mutation alleles. Indel mutations generated at a target site within an exon can lead to frame shift mutations in one or both alleles. One major advantage of the CRISPR–Cas9 system, as compared to conventional gene targeting and other programmable endonucleases, is the ease of multiplexing, where multiple genes can be mutated simultaneously simply by using multiple sgRNAs each targeting a different gene (Wang et al. 2013a, b). In addition, when two sgRNAs are used flanking a genomic region, the intervening region can be deleted or inverted (Blasco et al. 2014; Canver et al. 2014; He et al. 2015). Furthermore, chromosomal translocation can also be achieved by using two sgRNAs targeting two genomic loci located on different chromosomes (Choi and Meyerson 2014).

When a DSB is generated and a donor DNA template is provided, precise genetic modification can be introduced through the HDR pathway (Fig. 1). For small modifications, including incorporation of point mutations, defined indel mutations, as well as insertion of a short sequence such as a loxP site or an epitope tag, single-stranded oligodeoxynucleotide (ssODN) can be used as donor DNA. In this design, donor ssODN is designed to carry homologous sequences flanking the mutation and total size can be up to 200 nt. HDR efficiency does not appear to be directly correlated with donor homology lengths (Yang et al. 2013b), and HDR efficiency variation is likely due to the nature of the target genomic loci, which is still poorly understood. When DNA of larger sizes is to be introduced into a target site, a double-stranded donor plasmid carrying the transgene flanked by homologous arms is used (Yang et al. 2013a).

Because of the ease of use, CRISPR–Cas9 system has swiftly become the most commonly used tool for efficient genome editing of bacteria, plants, cell lines, primary cells, and tissues. Impressively, direct introduction of CRISPR–Cas9 into the zygote leads to efficient genetic modification of the genome in early embryos, which when brought to term develop into genetically modified animals (Hwang et al. 2013; Li et al. 2013a,b; Wang et al. 2013a; Yang et al. 2013a; Hai et al. 2014; Niu et al. 2014). Depending on the experimental setup, different methods can be used to deliver CRISPR–Cas9 system. When used as a genome editing tool in cultured cells, either electroporation or transfection is often used to deliver a plasmid containing a ubiquitous promoter driving Cas9 and sgRNA expression (Cong et al. 2013; Mali et al. 2013b). The genome editing efficiency achieved is highly dependent on a number of variables including the actual transfection efficiency, genomic locus intended to be targeted, and cell types. For genetic engineering in animals, Cas9 mRNA or protein and the sgRNA (with or without donor DNA) are introduced into zygotes by microinjection (Li et al. 2013a,b; Wang et al. 2013a; Yang et al. 2013a; Hwang et al. 2013; Hai et al. 2014; Niu et al. 2014). Germline modification has also been achieved in mice by transfection of plasmids expressing Cas9 and sgRNA into spermatogonial stem cells. After development to spermatids and injection into oocytes (i.e., fertilization), germline transmission of the specific genetic modification was achieved (Wu et al. 2015). Lastly, somatic cell genomic modification in mice has been achieved, by hydrodynamic tail vein injection of plasmids (Xue et al. 2014; Yin et al. 2014), as well as by injecting adeno-associated virus (AAV) expressing CRISPR–Cas9 in brain (Swiech et al. 2015).

The wild-type *S.pyogenes*-Cas9 (SP-Cas9) endonuclease has two nuclease domains, HNH and RuvC-like, each capable of cleaving one of the double-stranded target DNA when associated with a sgRNA (See Fig. 1). When either one of these domains is mutated, the Cas9-sgRNA complex becomes a sequence and strand-specific nickase (Cas9n). When used with two sgRNAs in close proximity and targeting opposite DNA strands, this “dual” Cas9 nickase generates a DSB with defined overhangs. The more commonly used Cas9n is D10A, where the RuvC domain is mutated and generates 5′ overhang (Mali et al. 2013a; Ran et al. 2013). H840A Cas9n that generates a 3′ overhang has also been successfully applied to mouse model generation (Shen et al. 2014). Furthermore, when both nuclease domains are mutated eliminating all endonuclease activity, Cas9 becomes a programmable DNA binding protein (deadCas9 or dCas9). Guided by sgRNA, dCas9, when fused with different effector domains such as KRAB domain or VP64, can be directed to promoters and directly influence the level of gene transcription (Cheng et al. 2013; Gilbert et al. 2013; Konermann et al. 2014). By using various dCas9-effector fusions, it may be possible to epigenetically modify a specific locus leading to change in gene expression in vitro and in vivo.

In addition to SP-Cas9, several orthologous CRISPR–Cas9 systems from other species have been characterized and applied to genome editing in mammalian cells (Cong et al. 2013; Esvelt et al. 2013; Hou et al. 2013). Compared to SP CRISPR–Cas9 system, most of these orthologous systems have different PAM requirements and crRNA and tracrRNA sequences. Their development and application will greatly expand the sequence space amendable to CRISPR–Cas9 targeting. In addition, by recognizing different sgRNA backbones, Cas9 from different species can be used to perform different functions in the same cells, without interfering with each other (Esvelt et al. 2013). These developments will be useful for applications such as modulation of transcription networks and labeling of multiple genomic sequences for live cell imaging.

## Design of CRISPR/Cas9 guide sequence—achieving a high targeting efficiency and specificity

Specificity of the CRISPR–Cas9 system is defined by the 20 nt located at the 5′ end of the sgRNA, which interacts with the target DNA by Watson–Crick RNA–DNA base-pairing. Although highly specific, Cas9-sgRNA binding to the target DNA can tolerate sequence mismatches, leading to mutations in unintended genomic loci (“off-target” effect) (Fu et al. 2013; Hsu et al. 2013; Lin et al. 2014). The principal variables that impact specificity may include target sequence length and composition, concentrations of the Cas9 protein and the sgRNA. Although much needs to be understood to fully define these parameters in a specific targeting experiment, below we attempt to discuss current strategies and available software for the design of the guide RNA.

Rational design of CRISPR guide sequence aims to maximize occurrence of the desired genetic modification at the target site, while minimizing the extent of unintended mutations at off-target sites. To begin defining parameters affecting on-target efficiency, recent work investigated the effect of target sequence composition on targeting efficiency (Wang et al. 2013b; Doench et al. 2014). Both studies concluded that a high or low GC content in guide sequence leads to lower efficiency, while other variables may also impact the efficiency (Wang et al. 2013b; Doench et al. 2014). When a guide sequence capable of mediating efficient on-target cleavage has been identified, it should be assessed for potential off-target activities within the genome of interest. As discussed earlier, CRISPR–Cas9 targeting specificity is determined by a 20 nt guide sequence located at the 5′ end of the sgRNA, plus the PAM sequence adjacent to the target site located at the genomic locus. Mismatches between the guide sequence and target DNA are tolerated to certain extent, especially in the region distal to the PAM sequence (Jinek et al. 2012; Fu et al. 2013; Hsu et al. 2013). Therefore, whenever possible, a guide sequence that matches or is highly similar to multiple genomic loci should be avoided to prevent off-target effects that may lead to unintended and often undetected genetic modification in the genome of the cell or organism. To assist researchers with the design of CRISPR–Cas9 experiments, a growing number of software tools have become available for designing guide RNA and predicting off-target profiles (see Table 1).

**Table 1:** A non-exhaustive list of available web-based programs to assist in guide RNA design

Name	Web site link	Off-target screening species	CRISPR–Cas designs		Comments	References
			WT nucleases	Nickase		
ZiFiT	http://zifit.partners.org/ZiFiT/	Human, mouse, plus other major species including *C. elegans* and *E. coli*	Yes	Yes	Analysis of sequences up to 1 kb Provides comprehensive off-target analysis data. Number of mismatches up to 3 for target sequence ≥18 nt and up to 2 for targeting sequences <18 nt Allows an informed selection of guide The site can also be used to design ZFN and TALEN	Sander et al. (2007, 2010)
CRISPR Design Tool	http://crispr.mit.edu	Human, mouse, and other major species including pig and chicken	Yes	Yes	Analysis of sequences up to 250 nt Clear display of guide choices Weighted sum of off-target hit scores in target genome. The scoring scheme was experimentally derived which take into account positions of mismatches, mean pairwise distance between mismatches and total number of mismatches.	Hsu et al. (2013)
RGEN tools	http://rgenome.net/	human, mouse, and other major species including a number of plant species	Yes	No	Two components: Cas-OFFinder for finding off-target sites. Microhomology search for predicting potential indels. Users can specify the maximum number of mismatches (up to 10) Requires input of guide sequences; i.e., these need to be previously designed. Standalone version available (C++/OpenCL implementation) and can search for any number of mismatches	Bae et al. (2014a, b)
E-CRISP	http://e-crisp.org/E-CRISP/	Human, mouse, and other major species including a number of plant species and pathogens	Yes	Yes	Output includes useful graphics User can specify experimental goals, e.g., KO, N-terminal tagging, C-terminal tagging Enables annotation filtering (e.g., exclude CpG islands) Scoring system based on specificity, annotation, and efficiency	Heigwer et al. (2014)
CHOPCHOP	http://chopchop.rc.fas.harvard.edu	Human, mouse, and other major species including drosophila and medaka	Yes	No	Output includes useful graphics Guide RNA design ranked by GC content, presence of G at position 20; location of target on gene architecture Allows PAM variations Off-target search up to two mismatches	Montague et al. (2014)
sgRNA Designer	http://broadinstitute.org/rnai/public/analysis-tools/sgrna-design	Human and mouse only (Guide RNA design based on efficiency score, no off-target prediction)	Yes	No	Analysis of sequences up to 10 kb Uses experimentally defined scoring scheme for on-target sites based on a logistic regression classifier trained on 1841 sgRNAs targeting multiple genes; Standalone version (python) available	Doench et al. (2014)

All provide real-time analysis. This is a brief list used by the authors and does not mean to be an exhaustive list. The order is based on published date

Most of the current guide RNA design and off-target prediction tools rely on rules derived from earlier studies based on simple sequence matches/mismatches (Fu et al. 2013; Hsu et al. 2013) and are focused on optimizing computational time, resources, and providing additional features to assist users to design the experiment to meet their specific goals. In general, most of currently available tools allow mismatches of target sequences up to 3 or 4 nucleotides (Sander et al. 2007,2010; Hsu et al. 2013; Heigwer et al. 2014), and in a GPU-based implementation, up to 1–10 mismatches in the online version and up to any number of mismatches in the standalone version (Bae et al. 2014b). Recent studies have begun to collect more experimental data to generate better models for computational predictions. Using experimentally derived models, “sgRNA Designer” predicts on-target efficacy using a logistic regression classifier trained on >1000 sgRNAs targeting multiple genes and scores sgRNAs using position-specific weights for nucleotides and dinucleotides (Doench et al. 2014). “CRISPR Design Tool” incorporates the number of mismatches, position of mismatches, and pairwise distances of mismatches into its off-target scoring scheme, which was derived from a set of systematically designed experiments (Hsu et al. 2013). For guide RNA designs, some tools allow specification of experimental goals by users for different desired modifications (e.g., insertion of tags, disruption of protein domains, etc.) or allow the use of gene architecture annotation to assist guide RNA designs (E-CRISP, CHOPCHOP). While most of the tools are designed for SP-Cas9 with NGG or NAG PAM sequences (ZiFiT, CRISPR Design Tool, E-CRISP, sgRNA design tool), a few provide flexibility for PAM sequences to allow design of guide RNA for orthogonal Cas9 proteins with different PAM requirements (RGEN Tools, CHOP–CHOP). With more experiments investigating how parameters such as target sequence effect on sgRNA expression and folding, as well as epigenetic context of on-target and off-target sites, we foresee in the near future better software packages or updates to existing tools will become available and benefit researchers in the design of more efficient and specific gene editing experiments.

### Strategies for mitigating off-target effect

As eluded above, intelligent design of sgRNA guide sequence is still in its infancy. Below, we have listed the main approaches that can be used in conjunction with software systems listed in Table 1.

(i) Choose a guide sequence with minimal potential off-target sites as determined by genome-wide homology searches. Among the guides, choose those with off-targets’ mismatches concentrated at the PAM proximal part of the guide sequence, as these are less tolerated for Cas9 function (Jinek et al. 2012; Fu et al. 2013; Hsu et al. 2013).
(ii) Use a guide sequence of shorter lengths (e.g., 17–19 nt). Fu et al. demonstrated that shorter targeting sequence in the sgRNAs could reduce off-target effect significantly with only a slight reduction of on-target efficiency (Fu et al. 2014).
(iii) Use dual nickase strategy. With a pair of closely positioned sgRNAs, Cas9 nickase (D10A mutant) can introduce two adjacent single-stranded nicks, leading to a DSB with defined overhangs. This approach has been demonstrated to reduce off-target activity by 50- to 1500-fold in cell lines and to achieve gene knockout in mouse zygotes without sacrificing on-target cleavage efficiency (Ran et al. 2013; Shen et al. 2014).
(iv) Use dCas9-FokI strategy. Using a pair of sgRNAs with optimal spacing and orientation, dCas9 fused with Fok1 nuclease domain can form dimer and generate DSB, similar to the design of ZFN and TALEN. The specificity is significantly increased using this strategy (Guilinger et al. 2014; Tsai et al. 2014).
(v) Off-target identification and mitigation. In addition to computational prediction, several strategies have been developed to experimentally identify off-target mutations (Frock et al. 2015; Kim et al. 2015; Tsai et al. 2015; Wang et al. 2015). By genotyping these potential off-target sites, cell lines containing desired genetic modification but free of off-target mutations can be identified. In the case of animal models, breeding can be used to segregate the desired allele from the off-target mutant alleles.

Each of these strategies comes with its own advantages and limitations. Hence, when designing CRISPR–Cas9 experiments, it is important to understand the potential impact of unintentional off-target mutations and the need for mitigating them. For example, if CRISPR–Cas9 is to be used for clinical intervention, it is essential that off-target effect be minimized and its potential impacts understood and/or removed. If, however, the aim is to develop animal models, it is less of a concern, as founder animals will be backcrossed and unintended mutant alleles segregated. A possible simple strategy to avoid misinterpretation of data due to off-target effect is to develop genetically modified models using at least two independent sgRNAs with different guide RNA sequences.

## Brief outline of CRISPR–Cas9-meditated genome editing in mouse

To help understand the general process of CRISPR–Cas9-mediated genome editing, here we outline the *basic* strategy and considerations for generating mouse models using CRISPR–Cas9 system (Table 2).

**Table 2:** Outline of mouse model generation using CRISPR–Cas9

Week	Milestone	Tasks	NHEJ-based indel knockout model	HDR-based knock in model
0–1	Model design	Identify the gene and the region to be targeted	TARGET THE first exon shared by all mRNA isoforms aiming for generating frameshift mutation	Identify the precise region to introduce point mutation, loxP site, or transgene
		Design and validate genotyping strategies	Conventional PCR should to be validated	Conventional or long range PCR and at times Southern blot strategies should be incorporated into design and validated
		Sequence analysis in the region of interest and check for polymorphisms for strain of interest	When working with a strain of mice other than the reference strain, it is essential to have the region of interest sequenced, to identify potential polymorphisms, which could cripple guide RNA recognition and reduce HDR efficiency	
		Design sgRNA(s) using software screening against genome of interest	Examine off-target profile and select optimal guide RNA sequences; i.e., likelihood of a frameshift mutation, avoiding sequence with significant off-target matches. Consider use of paired nickase	Examine off-target profile. It is desirable to ensure that upon HDR, target sequence is rendered refractory to further modifications by CRISPR–Cas9. Consider use of paired nickase
		Order reagents for sgRNA synthesis and donor assembly	N/A	Design ssODN for HDR, centered around point mutation, Tag, loxP, or region to be modified. ssODNs for HDR should be of full length PAGE purified
1–2 (KO) 1–3 (KI with ssODN) 1–8 (KI with dsDNA plasmid)	Reagent Preparation	sgRNA and Cas9 mRNA synthesis and quality control	Cas9 mRNA and sgRNA can be synthesized using commercial kits. Cas9 mRNA or protein can also be purchased from vendors. The quality of the RNA samples needs to be confirmed by gel electrophoresis, particularly poly adenylation product	
		Donor provision	N/A	Homology arm lengths between 200 nt to 10 kb, unique in sequence and isogenic to the strain of interest. Donor plasmid can be assembled by molecular techniques or synthesized in its entity
2–12 (KO) 3–13 (KI with ssODN) 8–18 (KI with dsDNA plasmid)	Founder Generation	Inject CRISPR–Cas reagents into mouse zygote	Microinjection materials must to be free of protein, chemical carryovers, and of particulate matter	
			Cas9 mRNA or protein, sgRNA	Cas9 mRNA or protein, sgRNA and donor ssODN or plasmid
		Implant manipulated zygotes into pseudo pregnant animals	Develop to term. Founder mice may carry homozygous mutations and must be monitored closely for phenotypes	
		Identify founder mice at 2–3 weeks of age	Screen DNA isolated from tail biopsy for NHEJ or HDR events by PCR and sequencing. For transgene KI, use Long Range PCR and Southern blot for genotyping Note, mosaicism is very often seen in founder animals and they must be bred to determine actual germline event	
12–22 (KO) 13–23 (KI with ssODN) 18–28 (KI with dsDNA plasmid)	Germline transmission	Set up breeding of putative founders	Breed with WT mice of chosen strain to generate F1 s. Crossing between founders is not recommended, as founder may be mosaic, each carry a unique mutant sequence(s) and carry with it unique off-target profiles	
		Identify modified F1 offspring	Identify and sequence the event to *fully* characterize the nature of the allele, e.g., frame shift mutation, large deletion, or correct HDR event	

The table outlines the tasks with approximate time lines required for genome editing in mouse

For generating indel-based null allele, single sgRNA targeting slightly 3′ of ATG or the first coding exon shared by all mRNA isoforms may be a good idea in general. Small indels generated using a single sgRNA can be either in-frame or out-frame mutations. The “RGEN Tools” is designed to analyze sequence surrounding the DSB site for the likelihood of microhomology-mediated repair (MMR) and a guide sequence can be chosen to optimize the occurrence of frameshift mutations (Bae et al. 2014a).

Knock in models can be divided into two categories practically. With “small” alterations, the intended mutation, such as incorporation of a point mutation, tag, loxP site, can be accommodated into a donor ssODN, along with homology sequences flanking the mutation, for a total size of 200 nt which is the limit for current ssODN synthesis. For the larger alterations that could not be accommodated onto a ssODN, a dsDNA plasmid can be synthesized or assembled by molecular techniques, with homology arm lengths from a few hundreds bases to many kb. The timeline for generating these two types of models vary accordingly, as it usually takes only days to synthesize a ssODN, it takes significantly longer to generate dsDNA plasmid.

Genotyping of indel, SNP incorporation and small tag insertions can be accomplished by amplification of the region encompassing the intended mutation (~500 nt) by PCR, followed by sequencing, to identify founder mice. Founders generated by the CRISPR–Cas9 technology often are mosaic, carrying the NHEJ, HDR as well as any remaining wild-type alleles all in one mouse. To identify the successful HDR alleles among the other events, the mixture of PCR product should be cloned into a plasmid and individual clones sequenced to unequivocally confirm the presence of the HDR allele. For transgene insertion alleles generated from use of donor plasmid, long range PCR or Southern blot should be used to examine integrity of the junction regions between donor homology arms and the genomic locus. Of particular notice is the possibility of additional unintended mutations originating from the off-target effect. These may be screened and if positive, mitigated by further breeding.

## Current challenges and future development of CRISPR–Cas9

Although CRISPR–Cas9 has been proven powerful and widely applied, it is still a relatively new technology and there is much to be understood and improved.

### Improving specificity and efficiency of the CRISPR–Cas9 system

A critical need in CRISPR–Cas9-mediated genome editing is to minimize the risk of off-target damage. As discussed above, various strategies can be used to minimize potential off-target effects, including truncated guide RNA, dual nickase, dCas9-FokI, etc. However, as each guide sequence likely has a variable number of off-target sites, experimental data need to be generated and analyzed to understand the factors related to off-target effect. With the use of multiple recently established methods for detecting off-target mutations and accumulation of data (Frock et al. 2015; Kim et al. 2015; Tsai et al. 2015; Wang et al. 2015), we can expect more comprehensive models for off-target prediction based on all the experimental data.

It is known that efficiency of genome editing using site-specific nucleases varies widely depending on genomic context. This is thought to result from the combined effects of different genetic composition and epigenetic state for each particular locus. For example, DNA accessibility has been correlated with transcription factor binding, and recently with Cas9 protein binding (Kuscu et al. 2014; Wu et al. 2014). Various histone modifications have been correlated with transcription factor binding and chromosome structure (ENCODE Project Consortium et al. 2012; Kundaje et al. 2012; Wang et al. 2012). Due to the complexity of genetic sequences, epigenetic modifications and different genomic loci across different cell types, we currently still lack basic understanding of how the binding, catalytic activity, and ultimately the efficiency of the CRISPR–Cas9 system is affected. A better understanding of these phenomena will significantly enhance our ability to efficiently and accurately target genomic sequences across the genome.

### Animal model generation: the challenge of bigger and faster

One major limitation of current method in developing genetically modified animals is that the founder animals are often mosaic, carrying more than two alleles with each appearing at a certain frequency. This is potentially due to CRISPR–Cas9 activity occurring after the first cell division of the zygote. A better understanding of cell division and control of the timing of CRISPR–Cas9 activity, aiming at modifying the genomic locus strictly at the one cell stage, may allow creation of homozygote mutant mice that may be suitable for direct phenotypic analysis without the need for further breeding. Improvement in this aspect will shorten the timeline from model creation to phenotypic analysis. Elimination of mosaicism will be of particular importance for generating genetically modified large animals (e.g., non-human primate), which takes years to breed and often have small liter sizes.

CRISPR–Cas9-mediated genome editing has been used to generate mouse models carrying mutations in a single or multiple genes, as well as reporter and conditional alleles (Wang et al. 2013a; Yang et al. 2013a). However, one of the most impactful uses of a mouse model is genetic humanization that requires replacement of the mouse gene or gene cluster with its human ortholog. This is extremely challenging and time-consuming using gene targeting method (Lee et al. 2014), and has not been achieved using CRISPR–Cas9-mediated genome editing. The average size of a gene is about 50,000 nt and genes from a family can be localized in a specific genomic locus (for example, the immunoglobulin gene cluster resides in a region that occupies a few million nucleotides). Replacement of a few kb long fragment has been demonstrated in human iPS cells (Byrne et al. 2015). More needs to be learnt and explored using the CRISPR–Cas9 system to engineer the genome in larger scale.

### Exploiting the sgRNA backbone to maximize efficiency and expand utility of the CRISPR–Cas system

By introducing an A-U flip and extension of the stem loop structure into the trascrRNA portion of the sgRNA backbone, Chen et al. achieved an improved efficiency of gene repression and genomic loci labeling using the CRISPR–Cas9 system (Chen et al. 2013). Moreover, different stem loop structures recognized by RNA binding proteins have been engineered into the sgRNA backbone, which serve as a bait to recruit different effectors, for the purpose of gene activation and repression (Konermann et al. 2014; Zalatan et al. 2015). By using sgRNAs targeting different genomic loci, with different effector recruiting stem loops, the same dCas9 protein could perform different functions at different target sites, therefore allowing for functional multiplexing using one CRISPR–Cas9 system (Zalatan et al. 2015).

In addition to DNA target, Cas9 can also bind with high affinity to single-stranded RNA (ssRNA) targets (O’Connell et al. 2014), although validity of its in vivo application has yet to be demonstrated. There are orthologous CRISPR systems that naturally target RNA molecules (Hale et al. 2009, 2012), therefore would be exciting resources to explore for RNA editing.

Interest in the CRISPR–Cas9 technology as a genome editing tool has increased exponentially in the last three years. Since its debut in 2012, there have already been more than 1000 papers published with CRISPR as the key word. The collective work from the field has culminated in the development of many innovative applications and major breakthroughs have been achieved, including the establishment of whole genome loss of function and gain of function screen (Wang et al. 2013b; Konermann et al. 2014; Shalem et al. 2014), as well as the generation of the first non-human primate knockout model (Niu et al. 2014). It is tantalizing what the future may hold for the CRISPR–Cas9 technology, particularly in the area of gene therapy, but we can be assured that further improvement and development of the technology will deliver even greater achievements.
